# Pharmacological Chaperones Attenuate the Development of Opioid Tolerance

**DOI:** 10.3390/ijms21207536

**Published:** 2020-10-13

**Authors:** Youta Okuyama, Hisayo Jin, Hiroshi Kokubun, Tomohiko Aoe

**Affiliations:** 1 Sanmu Medical Center, Department of Anesthesiology, 167 Naruto, Sanmu City 289-1326, Japan; dopetoxin@yahoo.co.jp; 2 Department of Anesthesiology, Chiba University Graduate School of Medicine, 1-8-1 Inohana, Chuo-ku, Chiba City 260-8670, Japan; jinhy@chiba-u.jp (H.J.); hiro_kokubun127@yahoo.co.jp (H.K.); 3 Pain Center, Teikyo University Chiba Medical Center, 3426-3 Anesaki, Ichihara City 299-0111, Japan

**Keywords:** opioid misuse, opioid tolerance, morphine, ER stress, unfolded protein response (UPR), pharmacological chaperone, glycogen synthase kinase 3β, BiP

## Abstract

Opioids are potent analgesics widely used to control acute and chronic pain, but long-term use induces tolerance that reduces their effectiveness. Opioids such as morphine bind to mu opioid receptors (MORs), and several downstream signaling pathways are capable of inducing tolerance. We previously reported that signaling from the endoplasmic reticulum (ER) contributed to the development of morphine tolerance. Accumulation of misfolded proteins in the ER induced the unfolded protein response (UPR) that causes diverse pathological conditions. We examined the effects of pharmacological chaperones that alleviate ER stress on opioid tolerance development by assessing thermal nociception in mice. Pharmacological chaperones such as tauroursodeoxycholic acid and 4-phenylbutyrate suppressed the development of morphine tolerance and restored analgesia. Chaperones alone did not cause analgesia. Although morphine administration induced analgesia when glycogen synthase kinase 3β (GSK3β) was in an inactive state due to serine 9 phosphorylation, repeated morphine administration suppressed this phosphorylation event. Co-administration of chaperones maintained the inactive state of GSK3β. These results suggest that ER stress may facilitate morphine tolerance due to intracellular crosstalk between the UPR and MOR signaling. Pharmacological chaperones may be useful in the management of opioid misuse.

## 1. Introduction

Opioids such as fentanyl and morphine are widely used as excellent analgesics for both acute pain (e.g., during surgery) and chronic pain (e.g., in cancer patients) [1,2]. However, the increases in addiction and overdose death due to opioid misuse arising from prescriptions made by medical institutions, especially in the United States where opioid analgesics have been heavily used in recent years, have become serious social problems. US government agencies have declared the opioid crisis a national emergency [3,4]. Chronic use of opioids induces tolerance that reduces their analgesic effects and opioid-induced hyperalgesia increases the painful sensation throughout the entire body [5], resulting in increased opioid doses, more addiction, and shortened life span [6,7]. About 16,000 deaths, or 36% of the 44,000 drug overdose deaths in the United States in 2013, were associated with prescribed opioids (2013 National Survey on Drug Use and Health). Approximately 9.9 million people aged 12 or older in 2018 misused prescription pain relievers, corresponding to 3.6% of the US population (2018 National Survey on Drug Use and Health).

Opioid tolerance develops due to multifaceted mechanisms such as altered intracellular signal transductions in sensory neurons, inflammation of neurons and glial cells, and reconstitution of neural circuits [8]. Opioids act via mu opioid receptors (MORs) expressed on the plasma membrane of primary sensory neurons, as well as various neurons in the cerebrum, brainstem, and dorsal horn of the spinal cord; opioid binding to MORs suppresses ascending nociceptive transmission and enhances descending pain inhibitory pathways, resulting in analgesia. MORs activate various signaling molecules through heterotrimeric guanine nucleotide-binding proteins (G proteins) [9], leading to an analgesic effect. MOR activation also induces G-protein-coupled receptor kinases to phosphorylate MORs [10,11], which can then be recognized by β-arrestins and internalized by clathrin-coated vesicles [12]. Transient uncoupling of MORs from signaling pathways due to their phosphorylation and subsequent intracellular trafficking causes opioid desensitization. β-arrestin-2 deletion enhances morphine analgesia and prevents the development of tolerance, but not dependence [12,13]. Most internalized MORs eventually return to the cell surface, resulting in re-sensitization [14,15,16]. Chronic morphine tolerance may accompany adaptations of the intracellular signal transduction of post-MOR activation, including increased protein kinase C activity [17] and up-regulation of N-methyl-D-aspartate receptor signaling [18,19,20,21]. Chronic morphine treatment also activates the glycogen synthase kinase 3β (GSK3β) and Src kinase pathways, while inhibition of these kinases has been shown to diminish morphine tolerance and restore analgesia [22,23,24].

Secretory and membrane proteins are inserted into the endoplasmic reticulum (ER) where their folding intermediaries interact with molecular chaperones such as immunoglobulin (Ig) heavy chain binding protein (BiP) [25]. Many physiological and pathological conditions, including those associated with increased secretory demands, ischemia, hypoxia, and genetic mutations, can cause aberrant protein folding and ER accumulation of misfolded proteins. These folding overloads lead to ER stress and initiate the unfolded protein response (UPR) [26,27], which increases ER quality control by reducing general protein synthesis, increasing ER chaperone levels and promoting ER-associated protein degradation. Chronic ER stress might also modulate intracellular signaling pathways, resulting in disorders such as type II diabetes [28], interstitial pneumonia [29], and neurodegenerative diseases [30,31].

We previously investigated whether ER stress could attenuate MOR signaling by examining the thermal antinociceptive effect of morphine in knock-in mice expressing mutant BiP [23]. BiP is an abundant chaperone present in the ER. A fraction of BiP proteins associating with unfolded proteins, are secreted from the ER [32]. At the Golgi complex, the Lys-Asp-Glu-Leu (KDEL) carboxyl terminal of BiP is recognized by the KDEL receptor, which facilitates the return of BiP to the ER via coat protein complex I (COPI) vesicles [33]. The KDEL-retrieval system is part of the proteostasis network [30]. The knock-in mice express mutant BiP with the KDEL amino acid sequence deleted, instead of normal BiP. Homozygous knock-in mice are born according to Mendelian law, while they died from neonatal acute respiratory distress syndrome on the first day after birth [34]. The heterozygous mutant BiP mice are alive and growing. Some of them revealed neurodegenerative disorders at a very old age [35,36]. The recognition of the KDEL-retrieval sequence by the KDEL receptor also modulates the signal transduction related to the UPR via activation of some kinases like mitogen-activated protein kinases [37,38]. Although repeated morphine administration caused tolerance in wild-type mice, mutant BiP mice showed less tolerance. These results suggest that ER stress and the ER chaperone BiP may be important contributors to morphine tolerance development. In this study, we examined the effects of pharmacological chaperones that attenuate ER stress on morphine tolerance in mice. The pharmacological chaperones, 4-phenylbutyric acid (PBA) and tauroursodeoxycholic acid (TUDCA) facilitate protein folding in the ER, and function as proteostasis regulators [39]. Their therapeutic effects on diverse pathologic states caused by ER stress have been shown in preclinical and clinical studies [40,41,42].

Higher opioid prescriptions have been shown to be at risk of increasing the frequency of opioid overdose-related complications [43]. In this study, opioid tolerance was induced in a mouse model by repeated use of morphine. We investigated whether the combined use of PBA and TUDCA with morphine suppressed the development of morphine tolerance. We also examined the expression of ER chaperones in neuronal cells expressing MORs and the activation status of GSK3β. Collectively, the results suggest the utility of using pharmacological chaperones to suppress an increase in the amount of prescription opioids.

## 2. Results

### 2.1. Repetitive Morphine Intake Induces Morphine Tolerance

**Figure 1:**
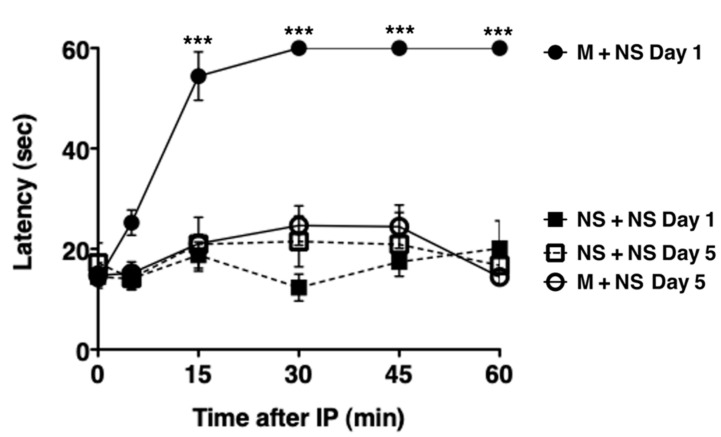
Tolerance development in mice following repetitive morphine intake. Mice received intraperitoneal morphine (M, 20 mg/kg) and oral saline (NS, 300 µL) twice a day for 5 days. IP represents an intraperitoneal injection of morphine. Hot plate tests were performed to evaluate analgesia after the first injection on day 1 and the tenth injection on day 5. The graph shows the response latencies (0–60 s) of mice on days 1 and 5 (*n* = 5). The response latencies after the first injection on day 1 were significantly longer than those after the tenth injection on day 5 at 15, 30, 45, and 60 min. *** *p* < 0.001, two-way analysis of variance with Bonferroni post-hoc test.

We evaluated morphine-induced antinociception by measuring response latencies in the hot plate test. Morphine tolerance was induced by intraperitoneal morphine injection twice a day for 5 consecutive days and assessed by hot plate tests after the first and tenth doses. Mice received intraperitoneal morphine (M, 20 mg/kg) and oral saline (NS, 300 µL) twice a day for 5 days. The first morphine injection on day 1 caused antinociceptive analgesia with the maximal latency (M + NS day 1, Figure 1, S1data). Repeated morphine injections significantly diminished the analgesic effect, producing minimal latency after the tenth morphine injection on day 5 (M + NS day 5, Figure 1). The response latencies before injections on days 1 and 5 (time 0 in Figure 1) were not significantly different. Thus, repetitive morphine intake for 5 consecutive days induced tolerance development.

### 2.2. Development of Morphine Tolerance Was Attenuated by Pharmacological ER Chaperone Administration

**Figure 2:**
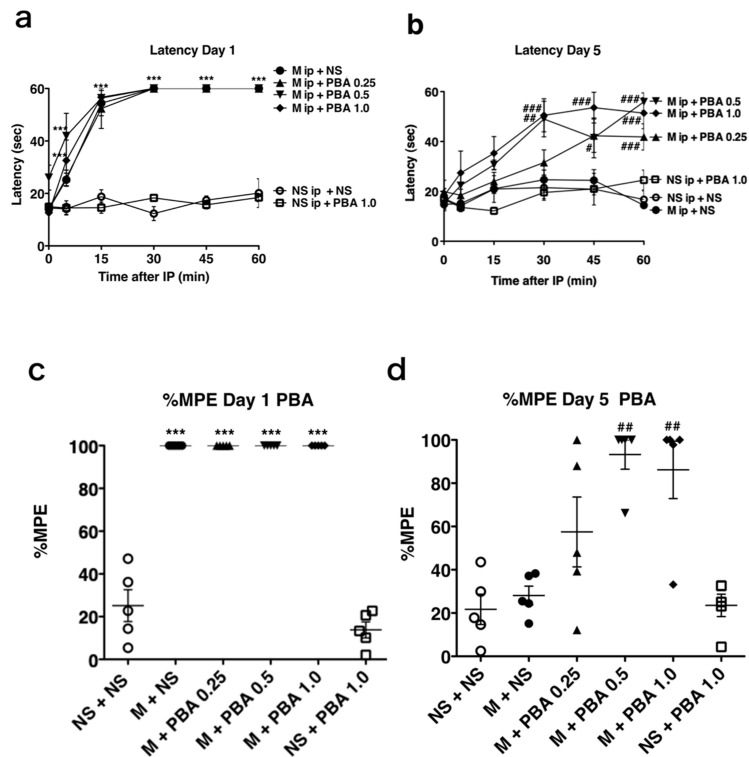
The pharmacological ER chaperone PBA attenuated morphine tolerance development. (**a**,**b**) Mice received intraperitoneal morphine (M, 20 mg/kg), and oral saline (NS, 300 µL) or 4-phenylbutyrate (PBA; 0.25, 0.5, 1.0 mg/kg) twice a day for 5 days. IP represents an intraperitoneal injection of morphine. Hot plate tests were performed to evaluate analgesia after the first injection on day 1 and the tenth injection on day 5. The graphs represent the response latencies (0–60 s) of mice on days 1 (**a**) and day 5 (**b**) (*n* = 5). *** *p* < 0.001; Values significantly higher than that in NSip + NS. ### *p* < 0.001, ## *p* < 0.01, # *p* < 0.05; Values significantly higher than that in Mip + NS. Two-way analysis of variance with Bonferroni post-hoc test. (**c**,**d**) The graphs represent the %MPE distribution of the mice on days 1 (**c**) and 5 (**d**). %MPE is described in Materials and Methods. By definition, the single longest response latency can occur in any time points. The mean %MPE of mice treated with intraperitoneal morphine and oral saline (M + NS) was significantly greater than that of mice treated with saline (NS + NS) on day 1 (**c**), but not on day 5 (**d**). The mean %MPE values on days 1 and 5 for mice treated with intraperitoneal morphine and oral PBA (M + PBA 0.5, 1.0) were significantly greater than those for mice treated with saline (NS + NS). *** *p* < 0.001; Values significantly higher than that in NS + NS. ## *p* < 0.01; Values significantly higher than that in M + NS. One-way analysis of variance with Bonferroni post-hoc test.

We evaluated the effect of the pharmacological ER chaperone PBA on antinociception with the hot plate test. Neither oral administration of 1.0 mg/kg PBA once on day 1 (NS + PBA 1.0, Figure 2a) nor twice daily administration for 5 days (NS + PBA 1.0, Figure 2b) was associated with analgesic effects, as demonstrated by minimal response latency on the hot plate test. On day 1, the response latencies of mice after the first morphine injection were significantly longer than those of mice receiving saline at 5, 15, 30, 45, and 60 min. We assessed the effect of PBA on morphine tolerance development. Co-administration of oral PBA with intraperitoneal morphine injection on day 1 did not affect the analgesic effect of morphine (Figure 2a). After co-administration of saline and morphine for 5 consecutive days, the response latencies did not become longer, indicating that tolerance had been induced (M + NS, Figure 2b). However, after co-administration of PBA and morphine for 5 consecutive days (M + PBA 0.25, 0.5, 1.0, Figure 2b), hot plate test response latencies were significantly longer than those after administration of morphine and saline for 5 days (M + NS, Figure 2b). On day 1, the mean maximum possible effects (%MPEs) of mice treated with morphine and PBA after the first injection were not significantly different from those of mice treated with morphine and saline (Figure 2c), but they were significantly greater on day 5 (M + PBA 0.5, 1.0, Figure 2d). The %MPE of mice treated with morphine for 5 days (M + NS, Figure 2d) became as low as that observed with saline treatment (NS + NS, Figure 2d). PBA alone did not have analgesic effects (NS + PBA 1.0, Figure 2c,d). Collectively, the results indicate that PBA attenuated tolerance development and preserved morphine’s analgesic effects.

**Figure 3:**
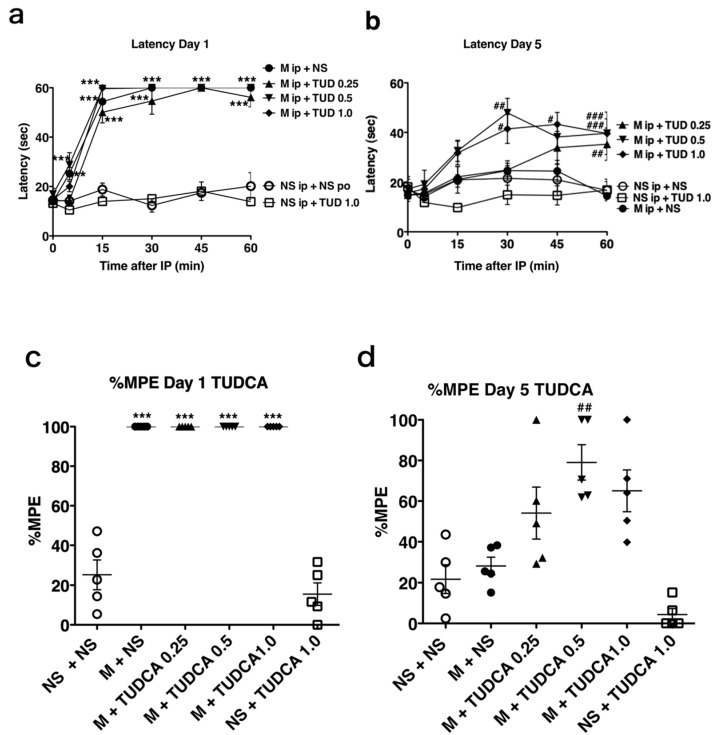
The pharmacological chaperone TUDCA attenuated morphine tolerance development. (**a**,**b**) Mice received intraperitoneal morphine (M, 20 mg/kg) and oral saline (NS, 300 µL) or tauroursodeoxycholic acid (TUD; 0.25, 0.5, 1.0 mg/kg) twice a day for 5 days. IP represents an intraperitoneal injection of morphine. Hot plate tests were performed to evaluate analgesia after the first injection on day 1 and the tenth injection on day 5. The graphs represent the response latencies (0–60 s) of the mice on days 1 (a) and 5 (**b**) (*n* = 5). *** *p* < 0.001, ** *p* < 0.01; Values significantly higher than that in NSip + NS. ### *p* < 0.001, ## *p* < 0.01, # *p* < 0.05; Values significantly higher than that in Mip + NS. Two-way analysis of variance with Bonferroni post-hoc test. (**c**,**d**) The graphs represent the %MPE distribution of mice on days 1 (**c**) and 5 (**d**). %MPE is described in Materials and Methods. By definition, the single longest response latency can occur in any time points. The mean %MPE of mice treated with intraperitoneal morphine and oral saline (M + NS) was significantly greater than for mice treated with saline (NS + NS) on day 1, but not on day 5. The mean %MPE values on days 1 and 5 were significantly greater for mice treated with intraperitoneal morphine and oral TUDCA (M + TUDCA 0.5, 1.0) than for mice treated with saline (NS + NS). *** *p* < 0.001; Values significantly higher than that in NS + NS. ## *p* < 0.01; Values significantly higher than that in M + NS. One-way analysis of variance with Bonferroni post-hoc test.

We also examined the effect of another ER pharmacological chaperone, TUDCA. On day 1, the response latencies of mice after the first morphine injection (M + NS, Figure 3a) were significantly longer than those of the mice receiving saline (NS + NS po) at 5, 15, 30, 45, and 60 min. The response latencies of mice after the tenth morphine injection (M + NS po) or TUDCA alone (NS + TUD 1.0) on day 5 were not significantly longer than those of control mice (intraperitoneal and oral saline only, NS + NS, Figure 3b).

However, on day 5, the response latencies of mice treated with morphine injection and oral TUDCA (M + TUD 0.25, 0.5, 1.0) were significantly longer compared with control mice (NS + NS, Figure 3b). TUDCA alone did not produce analgesia on the hot plate test (NS + TUD 1.0, Figure 3a,b, NS + TUDCA 1.0, Figure 3c,d), but co-administration of TUDCA and intraperitoneal morphine for 5 days (M + TUD 0.25, 0.5, 1.0, Figure 3b) induced significantly longer response latencies compared with morphine and normal saline (M + NS, Figure 3b). The mean %MPEs of mice treated with TUDCA and morphine (M + TUDCA 0.5, Figure 3d) were significantly greater than those of mice treated with morphine and saline on day 5 (M + NS, Figure 3d), indicating that TUDCA also attenuated the development of tolerance and preserved morphine’s analgesic effects.

**Figure 4:**
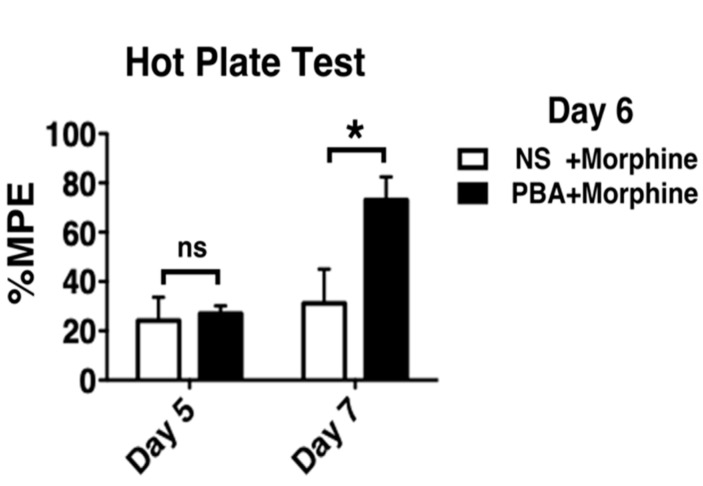
The pharmacological ER chaperone PBA restored morphine analgesia. Morphine (20 mg/kg) was administered intraperitoneally twice a day for 5 consecutive days. On day 6, intraperitoneal morphine (20 mg/kg) and oral normal saline (NS) was then administered twice in in the NS + Morphine (*n* = 5) group, while intraperitoneal morphine and oral PBA (1 g/kg) was administered twice in the PBA + Morphine (*n* = 5) group on day 6. Hot plate testing was performed after the tenth drug administration on day 5 and after intraperitoneal morphine injection on day 7. %MPE is described in Materials and Methods. By definition, the single longest response latency can occur in any time points. * *p* < 0.05, two-way analysis of variance with Bonferroni post-hoc test. ns, not significantly different.

We then assessed pharmacological chaperone effects after tolerance had developed. Morphine tolerance was induced by intraperitoneal morphine injection twice a day for 5 consecutive days, and confirmed by hot plate tests on day 5 (Figure 4, S2data). On day 6, those mice were administered with morphine and saline (NS) or PBA twice daily. Hot plate testing on day 7 revealed that the mean %MPE of mice treated with PBA and morphine on day 6 was significantly greater than that of mice treated with saline and morphine (Figure 4). Mice receiving PBA and morphine on day 6 had restored morphine analgesia on day 7, while mice receiving saline and morphine on day 6 remained tolerant to morphine.

### 2.3. Repetitive Morphine Intake Induced ER Chaperone Expression

**Figure 5:**
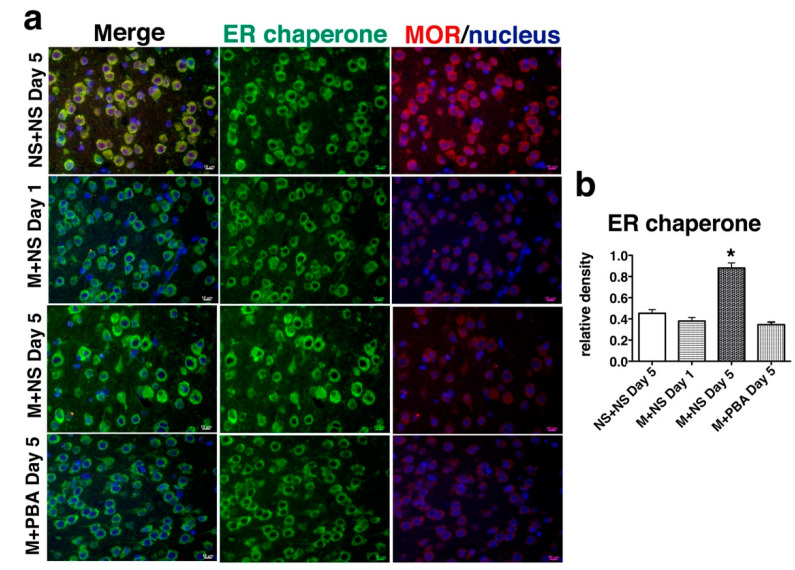
ER chaperone expression was enhanced in MOR-expressing neuronal cells in the PAG matter after repeated morphine administration. (**a**) Mice received intraperitoneal morphine (M, 20 mg/kg) and oral saline (NS, 300 µL) or 4-phenylbutyrate (PBA; 1.0 mg/kg) twice a day for 5 days. After the first injection on day 1 and the tenth injection on day 5, the brains were sectioned and double-immunostained with an anti-MOR antiserum (red) and anti-BiP monoclonal antibody (ER chaperone, green). Nuclei were stained with Hoechst 33258 (blue). Scale bars represent 10 µm. (**b**) MOR-immunopositive neurons in the PAG matter of mice that developed morphine tolerance after the tenth injection on day 5 (M + NS day 5) showed enhanced BiP expression compared with other mice. Densitometry was assessed in 10 cells in each group. * *p* < 0.001, one-way analysis of variance with Bonferroni post-hoc test.

MOR-expressing neurons in the periaqueductal gray (PAG) matter contribute to morphine tolerance [44]. To evaluate whether repetitive morphine administration affected ER function, we examined the expression of ER chaperones in MOR-expressing neuronal cells in the PAG region (Figure 5, S3data). After the first injection on day 1 and the tenth injection on day 5, mouse brains were sectioned and double-immunostained with an anti-MOR antiserum and anti-BiP monoclonal antibody (mAb) that recognizes the carboxyl KDEL sequence of BiP and other KDEL-containing ER chaperone proteins such as GRP94 [34]. MOR-immunopositive neurons in mice with tolerance after repetitive administration of morphine for 5 days (M+NS day 5; Figure 5) showed enhanced BiP expression compared with mice given a single injection on day 1 (M+NS day 1; Figure 5), suggesting that repetitive morphine administration induced ER stress. Co-administration of PBA and morphine for 5 days suppressed the enhanced expression of BiP, indicating that ER stress had been alleviated by the pharmacological chaperone (M+PBA day 5; Figure 5).

### 2.4. Pharmacological Chaperones Attenuate GSK3β Activation

**Figure 6:**
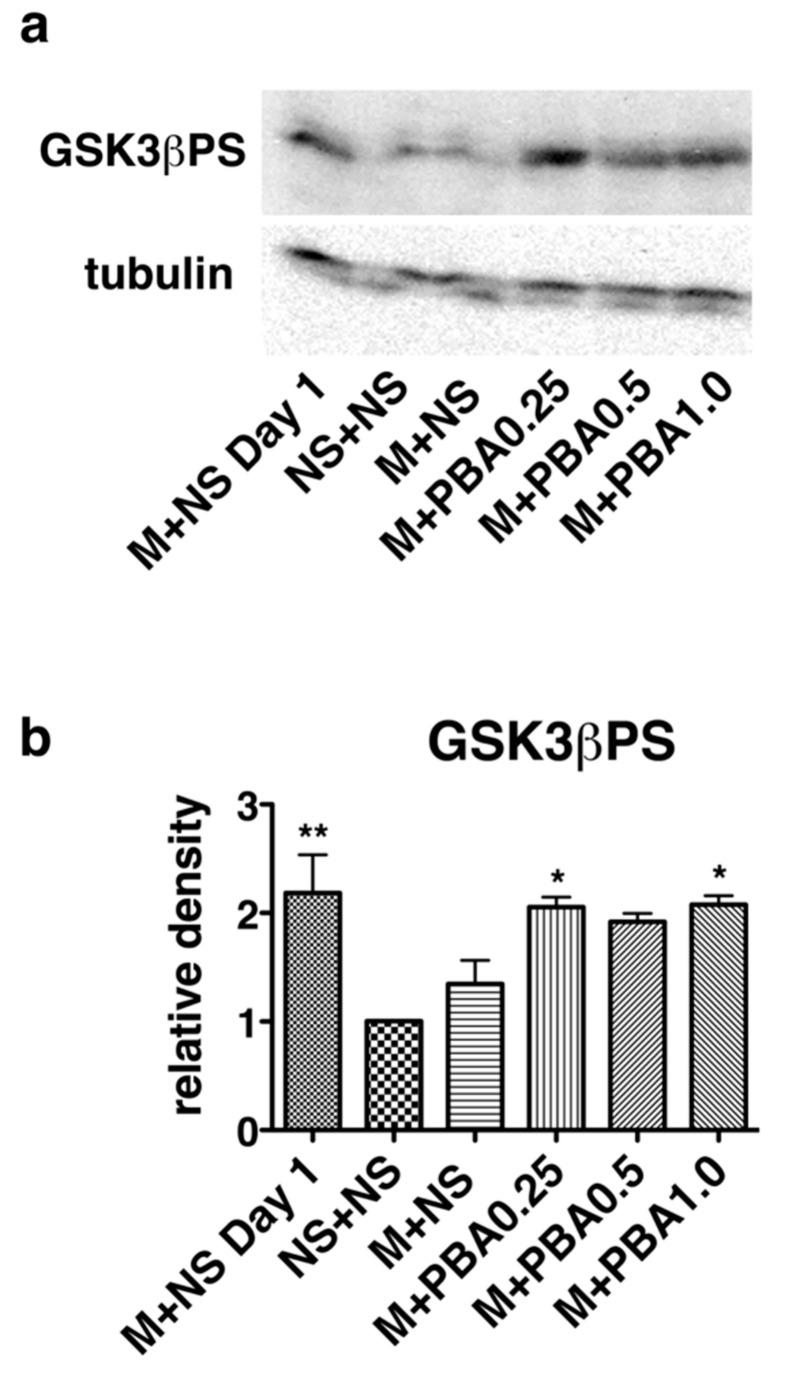
GSK3β activation was reduced in the mice treated with morphine and pharmacological ER chaperones. Mice received intraperitoneal morphine (M, 20 mg/kg) and oral saline (NS, 300 µL) or 4-phenylbutyrate (PBA; 0.25, 0.5, 1.0 g/kg) twice a day for 5 days. Brains were removed and section after the first injection on day 1 or the tenth injection on day 5. Brainstem expression levels of phosphorylated Ser9 of GSK3β (GSK3βPS) and tubulin were evaluated by western blotting (**a**). GSK3β inactivation was assessed by comparing the relative density (arbitrary units) of the GSK3βPS band to that of tubulin (**b**). The means ± standard error of the mean for three experiments are reported. The GSK3βPS values represent the relative values compared to those in mice treated with intraperitoneal saline and oral saline for 5 days (standardized as 1.0 in each experiment). The GSK3βPS values of mice given morphine and saline once (M+NS day 1) and mice receiving morphine and PBA (0.25, 1.0 g/kg) twice a day for 5 days were significantly higher than those of control mice (saline only; NS+NS). * *p* < 0.05, ** *p* < 0.01, one-way analysis of variance with Bonferroni post-hoc test.

MOR stimulation has been shown to inactivate GSK3β, which accompanies opioid analgesia. Repeated morphine administration leads to opioid tolerance formation and GSK3β activation, since inhibitors of this kinase alleviate the development of opioid tolerance [22,23]. Notably, ER stress activates GSK3β [45]. The serine/threonine kinase activity of GSK3β is regulated by the protein’s phosphorylation status. Phosphorylation of the Ser9 residue inactivate GSK3β kinase activity [46]. We evaluated GSK3β phosphorylation status in the brainstems of wild-type mice by performing western blots with an antibody specific for phosphorylated Ser9 of GSK3β (Figure 6, S4data and S5data). After repeated intraperitoneal morphine injections for 5 days, the mice developed morphine tolerance, but co-administration of PBA preserved morphine’s analgesic effects (Figure 2). GSK3β Ser9 phosphorylation was induced after morphine administration on day 1, but it was suppressed on day 5. Co-administration of PBA with morphine maintained GSK3β inactivation as demonstrated by Ser9 phosphorylation (Figure 6).

These observations suggest that chronic MOR stimulation due to repeated morphine injections may induce ER stress and alter signal transduction, including GSK3β activation. This may lead to the development of morphine tolerance. In contrast, co-administration of pharmacological chaperones that reduce ER stress may preserve the analgesic effects of morphine, suggesting that there may be crosstalk between the UPR due to ER stress and MOR signaling.

## 3. Discussion

Our results confirm that repeated morphine administration can cause antinociceptive tolerance in wild-type mice. The pharmacological ER chaperones PBA and TUDCA alleviated the development of morphine tolerance, presumably because they attenuated ER stress. Moreover, we showed that pharmacological chaperones restored opioid analgesia in mice after morphine tolerance had been induced.

Persistent accumulation of misfolded proteins beyond the capacity of ER quality control causes organelle stress, facilitating the UPR to expand capacity to deal with ER protein overload [26,47]. The ER transmembrane proteins, activating transcription factor 6 (ATF6), inositol requiring enzyme-1 (IRE1), and PKR-like ER kinase (PERK), are associated with BiP in the resting state, while BiP dissociates from them and target accumulated misfolded proteins for proper folding or degradation [27]. COPII (coat protein complex II) transport vesicles deliver ATF6 to the Golgi apparatus where it is cleaved [48]. The amino-terminal portion of ATF6 functions as a nuclear transcriptional factor to enhance the gene expression of proteins required for protein quality control such as ER molecular chaperones [49]. IRE1 and PERK bind to become homo-oligomers and are auto phosphorylated. IRE1 activation leads to regulated IRE1-dependent decay, then the cytoplasmic portion of the protein mediates *XBP1* mRNA splicing [50]. XBP1 protein functions as a transcriptional factor to enhance gene expression for the UPR [51]. Activated IRE1 also induces the activation of various intracellular signaling molecules such as Src kinase [45] and c-Jun N-terminal kinase [52]. Meanwhile, PERK activation induces the phosphorylation of eukaryotic translation initiation factor 2α (eIF2α) that suppresses protein translation, as well as induces ATF4 and C/EBP homologous protein activation, leading to cell death [53]. The UPR preserves the ER’s protein folding ability by enhancing the expression of ER chaperones, accelerating ER-associated degradation of misfolded proteins, and suppressing further protein synthesis.

Persistent overload of misfolded proteins causes a diverse array of disorders due to impaired functional protein synthesis and cell death [54,55], including neurodegenerative disease [30], dilated cardiomyopathy [56], and renal disease [57]. Another distinct mechanism by which ER stress causes human disease is that the UPR alters signaling pathways required for important cellular functions [38]. Obesity causes ER stress that induces the UPR, which may attenuate insulin receptor signaling through hyperactivation of c-Jun N-terminal kinase and serine phosphorylation of insulin receptor substrate-1. Crosstalk between the UPR and insulin receptor signaling has been shown to cause insulin resistance in type II diabetes [28]. Of more relevance to this research, chronic morphine administration may alter signal transduction due to persistent MOR activation [58]. In addition, MOR signaling may induce the UPR via calcium (Ca^2+^) kinetics, and the ER is the main store of Ca^2+^. MOR activation induces the ER to release Ca^2+^ into the cytoplasm [59]. ER chaperones including BiP are Ca^2+^-binding proteins, and the release of Ca^2+^ may disturb protein folding and induce the UPR. It has been shown that ER stress activates Src kinase [45] and GSK3β [60,61]. MOR-signaling-induced activation of these kinases has been associated with tolerance formation [22,23,24]. GSK3β plays important roles in a variety of human disorders, including inflammation, Alzheimer’s disease, mood disorders, diabetes, and cancer [62]. Thus, a mechanism similar to that occurring in type II diabetes might underlie the crosstalk between the UPR and analgesic signal transduction through MORs.

Pharmacological chaperones such as PBA and TUDCA have been shown to alleviate ER stress in cells and animal models [41]. Treatment with these compounds can normalize insulin actions in obese mice [63] as well as humans with obesity [64]. TUDCA has been shown to ameliorate the symptoms of patients with amyotrophic lateral sclerosis [65]. TUDCA is a taurine conjugate of ursodeoxycholic acid (UDCA), which promotes bile acid secretion and exerts a hepatocyte-protective effect. Clinical use of UDCA has been approved by the U.S. Food and Drug Administration (FDA) to treat primary biliary cholangitis. UDCA and TUDCA are major components of human bile acids. They are potent inhibitors of apoptosis because of multiple roles including interference upstream of the mitochondrial cell death pathway, inhibition of oxygen radical production, reduction of ER stress, and stabilization of the UPR [66]. PBA is a short-chain fatty acid that is naturally produced by colonic bacteria fermentation. The effects of PBA are due to its ability to regulate gene expression by acting as a histone deacetylase inhibitor and because it contributes to stabilizing protein conformation by serving as a proteostasis regulator [39,40,67]. PBA has been FDA-approved for urea cycle disorders. These two compounds have different chemical structures but similarly suppressed MOR tolerance formation, possibly via alleviating ER stress.

Opioids such as oxycodone and fentanyl have been prescribed for chronic pain, but the efficacy of long-term therapy has not been demonstrated [68,69,70]. High doses of opioid preparations for chronic pain can cause unfavorable side effects such as tolerance, hyperalgesia, addiction, and even death [71,72,73]. Buprenorphine, methadone, and naltrexone are currently used to reduce opioid use [73,74]. Buprenorphine and methadone are less preferred options because they themselves are opioids. Here we show that PBA and TUDCA ameliorate opioid tolerance and maintain morphine’s analgesia. Moreover, the analgesic effect of opioids could be recovered by pharmacological ER chaperone administration even after tolerance had been induced. Both PBA and TUDCA have few clinical side effects and may be effective treatments for opioid misuse through the reduction of opioid usage.

## 4. Materials and Methods

### 4.1. Animals

This study was carried out in accordance with the recommendations of the guidelines for animal experiments of Chiba University. The protocol was approved by the Institutional Animal Care Committee of Chiba University, Chiba, Japan (permission code; 28-204, 17 March 2016, 29-302; 3 March 2017). We used C57BL/6 male mice (20–25 g body weight, 10–15 weeks old) that had *ad libitum* access to food and water before the experiment.

### 4.2. Antibodies and Reagents

The following antibodies were used: rabbit polyclonal antibody against MOR-1 (AB1562 Chemicon, Temecula, CA, USA), mouse mAb against γ-tubulin (Sigma-Aldrich, St. Louis, MO, USA), mouse mAb SPA-827 against BiP (KDEL sequence; Stressgen, Victoria, BC, Canada), rabbit antiserum against GSK3β with phosphorylated Ser9 (sc-11757, Santa Cruz Biotechnology, Dallas, TX, USA), donkey anti-mouse IgG Alexa Fluor^®^ 488 (Invitrogen, Carlsbad, CA, USA), and donkey anti-rabbit IgG Alexa Fluor^®^ 555 (Invitrogen). The following reagents were used: Hoechst 33258 (B-1155, Sigma-Aldrich), morphine hydrochloride (Takeda Pharmaceutical Co., Tokyo, Japan), tauroursodeoxycholic acid, sodium salt (TUDCA; Calbiochem, San Diego, CA, USA), and sodium 4-phenylbutyrate (PBA; Enzo BML-EI320-0001, Farmingdale, NY, USA).

### 4.3. Immunohistochemistry

Immunohistochemistry was performed based on our laboratory protocols [23]. Brain sections were double-immunostained with an anti-MOR antiserum and anti-BiP monoclonal antibody, and then stained with a mixture of donkey anti-mouse IgG Alexa Fluor^®^ 488 and donkey anti-rabbit IgG Alexa Fluor^®^ 555. Immunolocalization was observed, and densitometry was done using ImageJ software (Wayne Rasband, National Institutes of Health, Bethesda, MD, USA). Integrated density of BiP staining in a cell with clear nuclear staining was measured.

### 4.4. Western Blotting

Western blotting was performed based on our laboratory protocols [23]. Imaging was obtained on an LAS-1000 equipped with Image Gauge™ software (Fuji Photo Film Co. Ltd., Tokyo, Japan). Densitometry was done using ImageJ software.

### 4.5. Hot Plate Test

The hot plate test was done based on our laboratory protocols [23]. The effects of treatment on the thermal nociceptive threshold were measured. Mice were placed on a 54.5 °C hot plate (Socrel model DS37; Ugo Basile, Gemonio, Italy), and the response latency to either a hind paw lick or jump was recorded. The animal was removed from the hot plate at 60 s to prevent serious tissue injury in the absence of a response, then a latency response of 60 s was assigned.

Morphine (20 mg/kg) dissolved in 300 µL of normal saline (M) or an equal volume of saline alone (NS) was administered intraperitoneally twice a day for five consecutive days. Each group was then orally administered either PBA (0.25, 0.5, or 1 g/kg dissolved in 300 µL of NS), TUDCA (0.25, 0.5, or 1 g/kg dissolved in 300 µL of NS), or saline (300 µL of NS) twice a day for 5 consecutive days. The hot plate test was performed after the first and tenth drug administrations on days 1 and 5, respectively for the experiments in Figure 1, Figure 2 and Figure 3. For the experiment in Figure 4, morphine (20 mg/kg) dissolved in 300 µL of normal saline was administered intraperitoneally twice a day for 5 consecutive days. Intraperitoneal morphine (20 mg/kg) and oral saline were then administered twice in the NS + morphine group, while intraperitoneal morphine and oral PBA (1 g/kg) were administered twice in the PBA + morphine group on day 6. Hot plate tests were performed after the tenth drug administration on day 5 and after intraperitoneal morphine injection on day 7.

Hot plate latency was measured at 5, 15, 30, 45, and 60 min after drug injection. Before drug administration, hot plate latency was measured three times, and the average was used as the pre-drug response latency at time 0 min. To analyze the effects of the drugs in the hot plate test, we calculated the %MPE as ([post-drug maximum response latency − pre-drug response latency]/[60-s cut-off time − pre-drug response latency]) × 100. The post-drug maximum response latency was defined as the single longest response latency.

### 4.6. Statistical Analysis

All data are expressed as mean ± standard error of the mean. One- and two-way analysis of variance were used, followed by Bonferroni post-hoc tests, to compare hot plate %MPE, latencies, and other values between groups. We used Prism 4.0 (GraphPad Software, San Diego, CA, USA) and considered *p* < 0.05 to indicate statistical significance.

## 5. Conclusions

Our results demonstrate that ER stress is significantly associated with the development of morphine tolerance in vivo. Pharmacological chaperones represent a promising therapeutic option for maintaining opioid analgesia without increasing prescriptions.

## Supplementary Materials

The following are available online at https://www.mdpi.com/1422-0067/21/20/7536/s1. S1data: The data of hot plate tests in Figure 1, Figure 2 and Figure 3. S2data: The data of hot plate tests in Figure 4. S3data: The data of densitometry in Figure 5. S4data: The data of densitometry in Figure 6. S5data: The western blots in Figure 6.

## Abbreviations

ATF4	Activating transcription factor 4
ATF6	Activating transcription factor 6
BiP	Immunoglobulin heavy chain binding protein
COP	Coat protein complex
eIF2α	Eukaryotic translation initiation factor 2α
ER	Endoplasmic reticulum
FDA	Food and Drug Administration
G proteins	Heterotrimeric guanine nucleotide-binding proteins
GSK3β	Glycogen synthase kinase 3β
IRE1	Inositol requiring enzyme-1
MORs	Mu opioid receptors
PBA	4-Phenylbutyric acid
PERK	PKR-like ER kinase
TUDCA	Tauroursodeoxycholic acid
UDCA	Ursodeoxycholic acid
UPR	Unfolded protein response
XBP1	X-box binding protein 1
